# Evidence-Informed Deliberative Processes – Early Dialogue, Broad Focus and Relevance: A Response to Recent Commentaries

**DOI:** 10.15171/ijhpm.2017.88

**Published:** 2017-07-22

**Authors:** Maarten P. Jansen, Rob Baltussen, Evelinn Mikkelsen, Noor Tromp, Jan Hontelez, Leon Bijlmakers, Gert Jan van der Wilt

**Affiliations:** ^1^ Radboud Institute for Health Sciences, Radboud University Medical Center, Nijmegen, The Netherlands.; ^2^ KIT (Royal Tropical Institute), Amsterdam, The Netherlands.; ^3^ Erasmus MC, University Medical Center Rotterdam, Rotterdam, The Netherlands.; ^4^ Institute of Public Health, Heidelberg University, Heidelberg, Germany.; ^5^ Africa Health Research Institute, Mtubatuba, South Africa.

The recent Health Technology Assessment international (HTAi) conference in Rome discussed how we can move ‘towards an integrated HTA framework for a more sustainable healthcare ecosystem’ and improve ‘the role of multi-stakeholder involvement in HTA to face ethical dilemmas for health system’s economic, social and environmental sustainability.’^1^

Evidence-informed deliberative processes (EDPs) to support priority setting for universal health coverage (UHC), as described in our editorial,^2^ are well-aligned with HTAi’s call for a more integrated HTA (or priority setting) framework. EDPs specifically achieve this by promoting early deliberation among a wide variety of stakeholders to identify, reflect and learn about the meaning and importance of values, informed by evidence on these values.^2,3^ Being a generic value-assessment framework, it may be applied to prioritization exercises with either broad or narrow scopes and should be contextualized to its local setting.

We agree with Gopinathan and Ottersen that the “focus of such processes [to support priority setting for UHC] should go beyond clinical services to accommodate also public health interventions.”^4^ We also concur with Lauer et al that priority setting should take into account higher level, systemic activities that can strengthen progression towards UHC such as ‘improving health-system governance,’ ‘ensuring equitable access to quality services,’ ‘separating prescribing from dispensing,’ or ‘setting up a pooled funding mechanism to purchase services.’^5^ Moreover, EDPs “should adapt to a diverse set of factors shaping the relationship between evidence and policy.”^4^ Ideally, EDPs are initiated at an early stage, as policy-relevant evidence can still be commissioned or searched for during this stage, and there is still time to reflect on input or ideas put forward by stakeholders. As Gopinathan and Ottersen point out, this requires involvement of the right stakeholders, including non-health stakeholders where relevant.^4^ At the same time, Lauer et al raise the question “who should be invited to the deliberative dialogue?”^5^ We argue that those affected by decisions should at least be provided the opportunity to participate and provide relevant reasons or evidence^3^ – and we acknowledge that it is hard to determine who the relevant stakeholders are, and that standardized approaches are required to identify and engage relevant stakeholders. The description by Gopinathan and Ottersen of the complex relationship between evidence and policy further demonstrates the challenge of knowledge translation and how evidence can eventually be presented so as to facilitate its uptake and inclusion in policy formulation or revision.^6^ More broadly speaking, these comments highlight the challenge of institutionalization: how to work towards a situation that priority setting for UHC gets integrated in the routine decision making processes at both national and sub-national levels.

With regard to whether stakeholder deliberation as part of EDPs departs from a ‘blank slate,’ we agree with Lauer et al that countries should not ignore global level policy as formulated and endorsed by the World Health Organization (WHO) member states.^5^ Universal goals do need to be aligned with local context and priorities. We also recognize the point made by Lauer et al that EDPs, generic as they are, are already in place to a certain degree at the country level and are supported by the WHO Secretariat,^5^ in the form of consultative groups, and part of health-sector reviews and strategic planning and budgeting. Likewise, as Chalkidou et al point out, the International Decision Support Initiative (http://www.idsihealth.org/) has been established to strengthen in-country institutional and technical capacity together with open participative processes for evidence-informed policy-making.^7^ We hope that such initiatives will benefit from the further development of EDPs.

That said, it is not our intention to devalue cost-effectiveness evidence in itself – or to criticize agencies specialized in producing this particular type of evidence.^5,7^ On the contrary, evidence on the cost-effectiveness of a clinical or public health intervention provides relevant information on the opportunity costs of alternative investments that are foregone. Hence we do not oppose, but instead welcome efforts to integrate evidence on multiple criteria as is the case in the extended cost-effectiveness analysis approach.^5,8,9^ Nevertheless, we agree with Chalkidou et al and would like to highlight again that “global approaches to CEA can hardly be too context-sensitive” for the very reason that “studies done by global players that ignore local contexts but nonetheless presume to advise may undermine local priorities and distort local spending decisions.”^7^ We furthermore applaud WHO for providing broader support and guidance to countries.^5^ Yet, we observe in practice that cost-effectiveness is often considered the dominant criterion when used in priority setting. This effectively makes the generation of other evidence and deliberation on additional criteria secondary to cost-effectiveness, which may undermine the legitimacy of decisions at the country level.

We thank all authors who commented on our editorial, and we look forward to working together in the coming years to help harmonize EDPs with like-minded initiatives and align them with sustainable country-led decision-making processes.

## Ethical issues

Not applicable.

## Competing interests

Authors declare that they have no competing interests.

## Authors’ contributions

MPJ wrote the first draft; all other authors contributed to revisions. All authors approved the final draft.

## Authors’ affiliations

^1^Radboud Institute for Health Sciences, Radboud University Medical Center, Nijmegen, The Netherlands. ^2^KIT (Royal Tropical Institute), Amsterdam, The Netherlands. ^3^Erasmus MC, University Medical Center Rotterdam, Rotterdam, The Netherlands. ^4^Institute of Public Health, Heidelberg University, Heidelberg, Germany. ^5^Africa Health Research Institute, Mtubatuba, South Africa.
